# Carbon and Nitrogen Isotopic Signatures as Metabolic Biomarkers of Nodal Metastasis and Recurrence in Oral Squamous Cell Carcinoma

**DOI:** 10.3390/cancers18091461

**Published:** 2026-05-01

**Authors:** Katarzyna Bogusiak, Zuzanna Popińska, Marcin Kozakiewicz, Piotr Paneth, Józef Kobos

**Affiliations:** 1 Department of Maxillofacial Surgery, Medical University of Lodz, 251 Pomorska, 92-209 Lodz, Poland; zuzanna.popinska@umed.lodz.pl (Z.P.); marcin.kozakiewicz@umed.lodz.pl (M.K.); 2 Institute of Applied Radiation Chemistry, Lodz University of Technology, 116 Żeromskiego, 90-924 Lodz, Poland; piotr.paneth@p.lodz.pl; 3 Department of Histology and Embryology and Department of Pathology, Medical University of Lodz, 251 Pomorska, 92-209 Lodz, Poland; jozef.kobos@umed.lodz.pl

**Keywords:** oral squamous cell carcinoma, stable isotope ratio mass spectrometry, δ^13^C, δ^15^N, metabolic signatures, lymph node metastasis, disease-free survival, penalized logistic regression, bootstrap validation

## Abstract

Background/Objectives: Oral squamous cell carcinoma (OSCC) exhibits substantial biological heterogeneity, and current clinicopathological risk stratification incompletely reflects tumor metabolic behavior. Stable isotope ratio mass spectrometry enables the quantitative assessment of carbon and nitrogen isotopic composition, potentially capturing cumulative metabolic reprogramming associated with tumor aggressiveness. This study evaluated whether isotopic signatures of tumor tissue and surgical margins are associated with lymph node metastasis and survival outcomes in OSCC. Methods: In this prospective study, 54 consecutive patients undergoing primary surgical treatment for OSCC were enrolled. Paired samples derived from tumor tissue and surgical margins were analyzed using isotope ratio mass spectrometry to determine the relative abundance of nitrogen-15 and carbon-13 isotopes. The primary endpoint was pathological lymph node metastasis. Secondary endpoints included disease-free survival and overall survival. Paired comparisons were performed using Wilcoxon signed-rank tests with false discovery rate correction. Logistic regression models for nodal metastasis were constructed using Firth penalization with bootstrap internal validation, while survival outcomes were evaluated using Cox proportional hazards models with model complexity restricted according to the number of events. Results: Tumor tissues demonstrated significantly lower δ^13^C and δ^15^N values and higher nitrogen-to-carbon ratios compared with surgical margins (all adjusted *p* < 0.05). In multivariable analysis, tumor δ^15^N was independently associated with lymph node metastasis and modestly improved model discrimination. However, it was not independently associated with disease-free or overall survival. Exploratory analyses indicated that higher δ^13^C values in surgical margins were independently associated with shorter disease-free survival. Conclusions: These findings suggest that isotope ratio mass spectrometry-based isotopic profiling identifies reproducible metabolic differences between tumor and margin tissues in OSCC. Tumor δ^15^N is associated with lymph node metastasis, whereas margin δ^13^C may reflect recurrence risk and potentially capture metabolic field effects. These findings are hypothesis-generating and warrant validation in larger, independent cohorts.

## Simple Summary

Oral squamous cell carcinoma is a common and aggressive cancer of the head and neck region. Current clinical and pathological factors do not fully reflect the biological behavior of the tumor. In this study, we investigated whether the natural abundance of stable carbon and nitrogen isotopes in samples derived from tumor and its surroundings can provide additional information about tumor metabolism and aggressiveness. By analyzing samples collected during surgery, we found consistent metabolic differences between cancer tissue and adjacent surgical margins. Nitrogen isotope abundance in tumor tissue was associated with the presence of lymph node metastasis, while carbon isotope composition in surgical margins was linked to the risk of disease recurrence. These findings suggest that isotope analysis may help identify patients at higher risk of aggressive disease and could complement existing prognostic tools in oral cancer research.

## 1. Introduction

Oral squamous cell carcinoma (OSCC) remains a clinically challenging malignancy characterized by a biological heterogeneity. Despite advances in surgical techniques, radiotherapy and systemic therapies, the five-year overall survival rate for OSCC is still low and hovers around 50–60%. Such poor prognosis can be related to the challenges in accurately predicting its propensity for local recurrences and nodal metastases [1,2]. Even though standardized staging and treatment protocols are routinely used, a substantial variability in long-term outcomes can still be observed. Recurrences occur in roughly one-third of patients, most often within the first two years of follow-up period. Local and regional relapse dominate the failure pattern, which contributes to poor salvage outcomes [3,4]. Cervical lymph node involvement, often occult at initial presentation, dramatically worsens prognosis by reducing survival by as much as 50% [5,6].

Current prognostic factors, based on clinical and pathological assessment, provide a valuable framework for risk stratification. They have been validated through numerous studies, including amongst others meta-analyses and retrospective research conducted on large cohorts [7,8,9]. Among these, the pathological TNM (pTNM) staging system remains the basis of risk stratification, consistently emerging as an independent predictor of disease-specific survival (DSS). Determining the pTNM category allows accurate stage assignment. These stages show clinically meaningful differences. Early-stage disease (stages I–II) is associated with disease-specific survival rates approaching 90%, whereas advanced stages (III–IV) demonstrate substantially poorer outcomes. Nevertheless, the pTNM system is inherently limited by its postoperative nature and partial overlap with other prognostic variables, which may reduce its utility for preoperative decision-making [10,11].

Another well-established clinical and pathological prognostic factor is the presence of regional lymph node metastasis. Its presence substantially increases the risk of mortality and recurrence, particularly the presence of extranodal extension (ENE). Although its prognostic relevance is well documented across large cohorts, diagnostic limitations persist, as conventional imaging fails to detect occult nodal disease [5,6,10,12]. Depth of invasion (DOI), especially values exceeding 5 mm, is closely associated with nodal metastasis, recurrence, and adverse survival. It has therefore been incorporated into contemporary staging systems. However, its independent prognostic value often diminishes in multivariable analyses [10,11,13].

Histopathological grade also remains a useful indicator of tumor aggressiveness, with poorly differentiated tumors exhibiting significantly worse outcomes. The main limitation of the histological grading system is its subjective nature and inter-observer variability that reduce reproducibility. Lymphovascular invasion (LVI) is a reliable adverse feature associated with reduced overall survival and increased risk of distant metastasis, whereas the prognostic impact of perineural invasion (PNI) appears less consistent [8,9,14,15]. Finally, the presence of distant metastasis has the most profound negative prognostic impact, although available evidence remains limited [16]. Molecular alterations, including EGFR overexpression, p53 mutations, and VEGF upregulation, further reflect aggressive tumor biology, yet their routine clinical application is limited by methodological variability and cost [3].

These commonly used prognostic tools have some limitations. They do not provide direct insight into tumor biological behavior and as a consequence in some cases the survival stratification can differ significantly. This uncertainty may lead to over- or undertreatment indicating a need for novel, more precise and prospectively validated biomarkers, reflecting metabolic reprogramming accompanying tumor progression [17,18].

As the search for reliable prognostic biomarkers intensifies, tumor metabolic profiling has emerged as a promising direction to explore. Increasing evidence suggest that metabolic pathway changes are a hallmark of cancer, playing a critical role in tumor progression, invasion and resistance to therapy. Stable isotope ratio mass spectrometry (IRMS) can be used to quantify natural variation in stable isotopes, particularly carbon-13 (^13^C) and nitrogen-15 (^15^N) abundances in biological samples. The isotopic ratios ^13^C/^12^C and ^15^N/^14^N are usually expressed in delta (δ) notation relative to international standards (PDB—Pee Dee Belemnite for carbon and AIR for nitrogen). Previous studies have demonstrated that isotopic signatures in malignant tissues altered compared with healthy controls, suggesting that isotopic composition may serve as an indirect marker of tumor metabolism.

By analyzing isotopic ratios (^13^C/^12^C) and (^15^N/^14^N) in tumor samples, IRMS can reflect cumulative effects of shifts in metabolic pathways, including amino acid turnover, enhanced glycolysis, lipid metabolism and nitrogen utilization, that can be indicative of increased cellular proliferation, which correlate with advanced disease stages and adverse histopathological features like angioinvasion and perineural invasion [19,20].

Although initial studies have shown that IRMS parameters do not independently predict lymph node metastasis in multivariate models—where factors such as age, gender, and clinical stage predominate—the technique’s ability to characterize tumor aggressiveness offers potential as a prognostic indicator for local and nodal recurrences, as well as overall survival. For instance, depleted δ^13^C values have been associated with metabolic shifts toward rapid growth and invasion, while enriched ^15^N levels in advanced-stage tumors suggest heightened protein catabolism, both of which may signal a higher risk of recurrence and poorer long-term outcomes [19].

While IRMS has been applied in several malignancies, data regarding its prognostic significance in OSCC remains limited. In particular, the potential relationship between isotopic heterogeneity within the tumor and at the tumor–margin interface and long-term clinical outcomes has not been fully explored.

The present study investigates whether isotopic parameters of carbon ^13^C and nitrogen ^15^N measurements in tumor and margin tissues are associated with overall survival and disease-free survival in patients with OSCC.

We hypothesized that isotopic signatures of tumor tissue, surgical margins, and tumor–margin differences independently predict lymph node metastasis and disease recurrence beyond established clinicopathological risk factors.

## 2. Materials and Methods

### 2.1. Study Design

This prospective study enrolled consecutive patients diagnosed with primary oral squamous cell carcinoma (OSCC) who underwent curative surgical treatment. Eligibility criteria included:

1. Histopathological confirmation of primary OSCC;
2. Availability of paired tumor tissue and corresponding surgical margin samples;
3. Treatment with radical surgical resection as the primary therapeutic approach;
4. Availability of complete and documented follow-up data.

Patients were excluded if they had received prior chemotherapy, presented with distant metastases at the time of diagnosis, or had incomplete clinical or follow-up information. Patients with malnutrition (BMI < 18.5) or with diabetes mellitus were not eligible for inclusion. The participants did not adhere to any dietary regimen. Clinical, pathological, and outcome data were prospectively collected for all included patients. Information on the isotopic abundance of ^15^N and ^13^C of tissue specimens derived from the tumor and its margin was obtained using IRMS procedure.

Overall survival (OS) was defined as the interval from the date of surgery to death from any cause. Disease-free survival (DFS) was calculated from the date of surgery to the first documented event of local, regional, or distant recurrence, or death, whichever occurred first.

Patients were monitored during routine follow-up visits scheduled at three-month intervals. Suspected local, regional, or distant recurrences were confirmed by histopathological evaluation and/or radiological imaging following completion of primary treatment.

The study protocol was approved by the local Bioethics Committee (RNN/185/18/KE). All patients provided written informed consent. The study was conducted in accordance with the Declaration of Helsinki.

### 2.2. Tissue Sampling and Preparation

Tumor specimens and surgical margin tissues (approximately 2 mm × 2 mm) were collected intraoperatively during primary tumor resection. All specimens were collected intraoperatively under standardized conditions. To minimize pre-analytical variability, including ischemia time effects, tissue samples were immediately processed and frozen at −70 °C according to a uniform protocol applied to all patients. In total, 8 tissue samples were extracted from each patient (4 from carcinoma infiltration and 4 from its margin). Margin samples were obtained from macroscopically normal-appearing tissue adjacent to the primary lesion at a targeted distance of 10 mm from the macroscopic tumor border. All margin samples paired for IRMS were histologically verified by a senior pathologist to be free of dysplasia and neoplastic infiltration. All collected tissues were processed according to standardized protocols for subsequent analyses.

Two samples from tumor and two from surgical margin were immersed in formalin, embedded in paraffin and assessed by an experienced pathologist (JK). Additionally, the entire postoperative tumor and lymph node specimens underwent routine histopathological assessment. The remaining 4 paired samples were frozen at −70 °C and prepared for IRMS analysis.

### 2.3. IRMS Procedure

After samples collection each of them was frozen at −70 °C for at least 48 h. Subsequently, they were lyophilized using a Christ Delta 1-24 LSC lyophilizer (GmbH, Osterode am Harz, Germany).

After lyophilization, tissue material was homogenized and portions of approximately 3 ± 1 mg were weighed into tin capsules for isotopic analysis. On average, three subsamples were analyzed per individual tissue section.

Combustion was performed in the presence of vanadium pentoxide as an oxidation catalyst. Thiobarbituric acid served as the working laboratory reference material and was calibrated against internationally accepted standards: atmospheric nitrogen for δ^15^N and Pee Dee Belemnite (PDB) for δ^13^C. Instrument calibration and quality control were ensured through repeated analyses of certified reference materials in accordance with standard IRMS validation procedures. Calibration reproducibility is a key aspect of ensuring the reliability and consistency of measurement results and process control. IRMS analysis is performed using the bracketing approach, with the following measurement sequence: standard-sample-standard. Equal delta values for the standards during the measurements confirm calibration reproducibility. The bracketing method reduces variability and ensures the consistent quality of the results.

Isotopic measurements were performed using a Sercon (Crewe, Great Britain) SL20–22 continuous-flow isotope ratio mass spectrometer coupled with a Sercon SL elemental analyzer, enabling simultaneous determination of carbon and nitrogen isotope ratios. Isotopic compositions were reported as δ values (‰) according to the following equation:

δX (‰) = (R_sample/R_standard − 1) × 1000

where X denotes either δ^13^C or δ^15^N, and R represents the ratio of the heavy to light isotope (^13^C/^12^C or ^15^N/^14^N). Carbon isotope values were expressed relative to the PDB standard, while nitrogen isotope values were referenced to atmospheric nitrogen. Analytical precision was verified using standard reference materials, yielding a measurement uncertainty of ±0.2‰ for δ^13^C and ±0.3‰ for δ^15^N.

Additionally, elemental composition parameters were recorded, including minimum and maximum mass percentages of carbon and nitrogen, median values with interquartile ranges, mean ± standard deviation, total mass percentages of C and N, and the nitrogen-to-carbon ratio ([N]/[C]).

### 2.4. Statistical Analysis

Continuous variables were summarized using medians and interquartile ranges; categorical variables as counts and percentages. Paired tumor–margin comparisons were performed using Wilcoxon signed-rank tests with bootstrap-derived 95% confidence intervals. False discovery rate correction was applied where appropriate. For lymph node metastasis (31 events), multivariable models were constructed using Firth penalized logistic regression, with the number of predictors restricted to preserve acceptable events-per-variable ratios and minimize overfitting. Model discrimination was assessed using AUC. Internal validation was performed using bootstrap resampling (1000 iterations) to obtain optimism-corrected AUC values. Calibration was evaluated using calibration plots and Brier scores. For survival analyses (20 DFS events), Cox proportional hazards models estimated hazard ratios per one standard deviation increase. Multivariable models were restricted to avoid overfitting. The number of predictors included in multivariable models was restricted according to the number of events to maintain an acceptable events-per-variable ratio and reduce the risk of overfitting. Proportional hazards assumptions were verified using Schoenfeld residuals. Kaplan–Meier curves were generated for visualization purposes only; primary inference relied on continuous modeling.

Exploratory analyses were pre-specified as hypothesis-generating and were intended to further characterize the internal structure of the spectrometric dataset and to screen additional biomarkers for potential prognostic relevance. First, an exploratory survival screening was performed for additional spectrometric parameters, including δ^13^C, percentage mass content of carbon and nitrogen, and the [N]/[C] ratio, assessed in tumor tissue, surgical margins, and tumor–margin differences. These analyses used Cox proportional hazards models with hazard ratios expressed per one standard deviation increase and were adjusted for selected clinicopathological covariates (depth of invasion and pathological T category; extranodal extension was additionally included where model stability allowed). False discovery rate correction was applied across exploratory biomarker screening analyses. For descriptive visualization only, Kaplan–Meier curves stratified by median biomarker values were generated.

To assess the internal relationships among spectrometric variables, Spearman rank correlation analysis was performed between tumor–margin differences in spectrometric biomarkers and selected clinicopathological variables. In addition, principal component analysis (PCA) was applied to standardized Δ(T–M) biomarkers to explore the dominant variance structure and potential separation according to nodal status. Unsupervised clustering was then performed on the standardized tumor–margin difference profiles to identify potential patient subgroups with shared metabolic patterns.

## 3. Results

### 3.1. Study Group

Fifty four consecutive patients in the age of 66.6 ± 9.2 years old, with oral squamous cell carcinoma (OSCC) that was treated surgically were included in the analysis. The cohort was predominantly male (33/54; 61.1%), with a mean age of 64.6 ± 9.3 years. The mean age of female participants were similar to that of male patients and equaled 69.6 ± 9.5 years. A history of smoking was reported by 35/54 (61.1%) patients, while alcohol consumption was documented in 16/54 (29.6%). Baseline clinicopathological characteristics of the study group are presented in Table 1.

The most common primary tumor location was the lower gingiva (21/54; 38.9%), followed by the floor of the mouth (18/54; 33.3%) and the tongue (12/54; 22.2%). Advanced disease was common, with the majority of patients (43/54; 79.6%) presenting with pathological stage IV disease, according to the pathological classification (AJCC, 8th edition). The most frequent tumor categories were pT4a (23/54; 42.6%) and pT3 (15/54; 27.8%). Regarding nodal status, pN3b (26/54; 48.1%) predominated followed by pN0 (22/54; 40.7%). Cervical lymph node metastases (N+) were identified in 31/54 (57.4%) patients, and extranodal extension (ENE) was present in 25/54 (46.3%) cases. Lymphovascular invasion and perineural invasion were observed in 20/54 (37.0%) and 17/54 (31.5%) patients, respectively. The median number of resected lymph nodes was 34 (IQR, 26–42). Depth of invasion (DOI) was frequently greater than 10 mm and was observed in 29 patients (53.7%).

The reported N+ status refers to pathological nodal involvement at the time of primary surgery (pN+). Regional recurrences during follow-up were analyzed separately within the DFS endpoint.

Postoperative radiotherapy was administered to 46/54 (85.2%) patients, while adjuvant chemotherapy was delivered to 6/54 (11.1%).

At the time of analysis, locoregional recurrence, distant metastases and death had occurred in a considerable number of patients, reflecting the aggressive clinical profile of the cohort. Local recurrences were observed in 16 (29.6%) cases, most commonly within the first years after treatment. Regional lymph node metastases occurred more frequently. They were identified in 31 (57.4%) patients, most commonly involving the ipsilateral cervical region. Distant metastases were present in a small substantial proportion of patients—13 (24.1%). They were most often located in the lungs or bones.

During follow-up, disease recurrence was observed in 20/54 (37.0%) patients, and death from any cause occurred in 35/54 (64.8%). The median overall survival (OS) was 205 weeks, which corresponded to approximately 47 months (almost 4 years). The median disease-free survival (DFS) was 181.5 weeks corresponded to approximately 42 months, that is about 3,5 years.

No statistical comparisons were performed for baseline characteristics.

### 3.2. Comparative Isotopic Profiling of Tumor Samples and Corresponding Surgical Margins 

**Figure 1:**
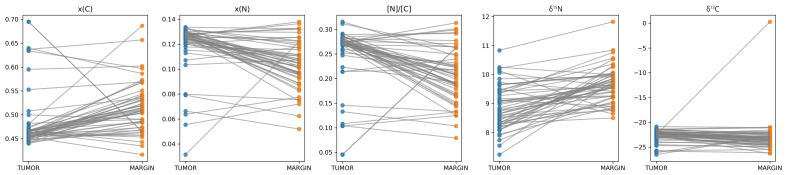
Paired spectrometric differences between tumor tissue and surgical margins.

Paired comparisons between tumor tissue and corresponding surgical margins revealed statistically significant differences across all five lead spectrometric biomarkers. Detailed data are presented in Table 2 and visualized using paired plots in Figure 1. Tumor tissue demonstrated significantly lower mean percentage mass content of carbon 13C compared with margins (46.2% versus 51.1%, respectively).

The median tumor–margin differences of carbon content equaled 4.2% and it was lower in tumor sample. The opposite pattern was noted for percentage mass content of nitrogen—it was significantly higher in tumor tissues. The nitrogen-to-carbon ratio was also significantly higher in tumor tissues.

In contrast δ^13^C and δ^15^N values were significantly lower in tumor samples. Namely, tumor samples exhibited δ^13^C and δ^15^N values that were significantly closer to the reference standard (PDB and AIR) than those measured in the margins.

Median tumor–margin differences were consistent in direction across the cohort, with narrow bootstrap-derived 95% confidence intervals. All paired comparisons remained statistically significant after false discovery rate correction. These findings indicate a reproducible and directionally consistent alteration of the spectrometric signature in tumor tissue relative to adjacent surgical margins.

### 3.3. Univariate Association of Spectrometric Biomarkers with Lymph Node Metastasis

Univariate analyses evaluating associations between spectrometric biomarkers and lymph node metastasis (N+) are presented in Table 3. Biomarkers measured in tumor tissue, surgical margins, and tumor–margin differences were assessed using Mann–Whitney U tests and univariate logistic regression models.

No biomarker demonstrated a statistically significant association with the presence of lymph node metastasis after correction for multiple testing. Several biomarkers, particularly nitrogen-related measures and tumor–margin differences, showed consistent but non-significant trends toward association with nodal spread when expressed per one standard deviation increase. Higher δ^13^C values of tumor–margin differences showed trend toward increased odds of nodal spread when expressed per 1 SD increase (OR = 1.70; 95%Cl: 0.59–4.89). Effect size estimates per one standard deviation were directionally coherent across analyses, although confidence intervals overlapped unity.

### 3.4. Multivariable Models for Lymph Node Metastasis

**Figure 2:**
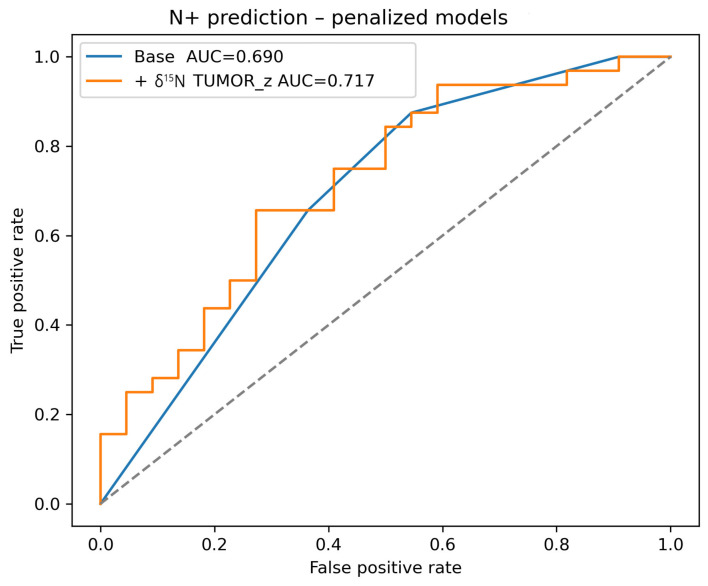
Receiver Operating Characteristic (ROC) curves for prediction of nodal metastasis (N+) using penalized models.

Multivariable penalized logistic regression models were constructed to evaluate whether spectrometric biomarkers provided information beyond established clinicopathological factors. The clinical base model, including depth of invasion and pathological T category, demonstrated moderate discrimination for lymph node metastasis (AUC = 0.690; Table 4, Figure 2). When biomarkers were added individually, δ^15^N measured in tumor tissue produced the largest improvement in discrimination (AUC = 0.717; ΔAUC = 0.027). Inclusion of tumor δ^15^N increased the model AUC and yielded a consistent increase in the odds of nodal disease per one standard deviation increase in the biomarker. Thus, although δ^15^N TUMOR was not significant in univariate analysis after multiple-testing correction, it contributed independently to the multivariable prediction of nodal metastasis. In multivariable analysis δ^15^N measured in tumor tissue emerged as an independent model-based predictor of lymph node metastasis. This suggests that it may capture metabolic information complementary to DOI and pT. The magnitude of this improvement was modest, and the finding should therefore be interpreted as hypothesis-generating. Tumor–margin differences of δ^15^N values provided limited additional prognostic information once clinicopathological factors were included.

### 3.5. Survival Analyses

Disease-free survival (DFS) and overall survival (OS) were analyzed using final endpoint definitions. DFS was defined as the time from first surgery to any disease recurrence (local, regional, or distant) or death, whichever occurred first. Overall survival was characterized as time from first surgery to death or last follow-up visit. The statistical evaluation was performed with δ^15^N measured in tumor tissues as the primary biomarker of interest.

**Figure 3:**
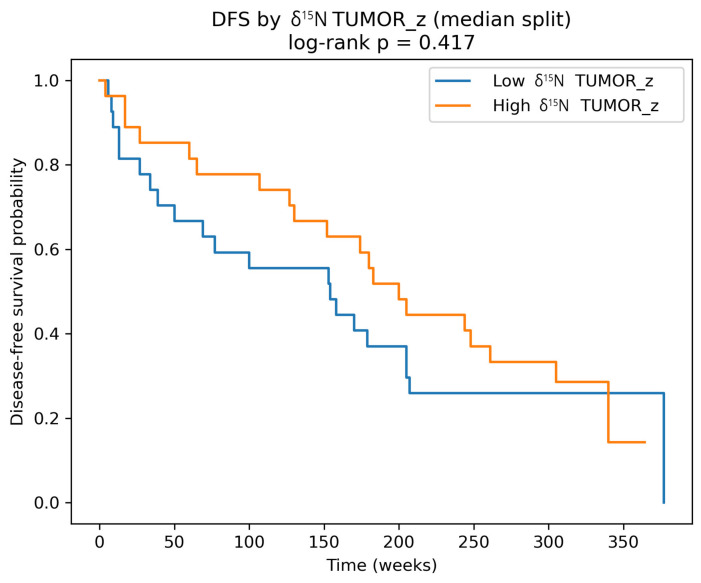
Tumor_z denotes 2-score standardized biomarker values measured in tumor tissues.

Kaplan–Meier analysis of DFS was performed using a median-based cut-off separation of survival curves; however, the difference did not reach statistical significance (Figure 3, log-rank *p* = 0.42).

In multivariable Cox proportional hazards models adjusted for depth of invasion, pathological T category, and extranodal extension, δ^15^N measured in tumor tissue was not independently associated with DFS (HR 1.18, 95% CI 0.85–1.63, *p* = 0.33). Depth of invasion (HR 0.43, *p* = 0.03) and pathological T category (HR 4.74, *p* = 0.01) remained the principal determinants of disease-free survival. Proportional hazard assumptions were satisfied for all models. The lack of separation in dichotomized Kaplan–Meier curves does not negate the adjusted continuous association; dichotomization was used only for visualization.

Analyses of overall survival yielded similar results. The δ^15^N showed no independent association with OS after adjustment for clinicopathological variables.

### 3.6. Exploratory Analyses

Exploratory analyses were performed to further characterize spectrometric biomarkers and to assess their potential relationships with clinical outcomes and underlying data structure. In an exploratory survival screening, the prognostic relevance of additional spectrometric parameters, including δ^13^C, total mass percentages of carbon and nitrogen as well as the [N]/[C] ratio, was evaluated across tumor tissue, surgical margins, and tumor–margin differences. These analyses were conducted using multivariable Cox proportional hazards models adjusted for depth of invasion and pathological T category.

**Figure 4:**
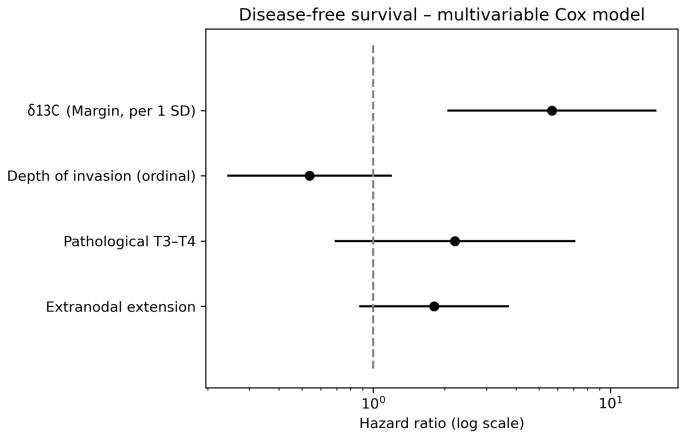
Multivariable Cox proportional hazards model for disease-free survival. Forest plot showing hazard ratios (HRs) with 95% confidence intervals for spectrometric and clinicopathological variables included in the model. δ^13^C in surgical margins was entered as a standardized variable (per 1 standard deviation increase). The model additionally included depth of invasion (ordinal), pathological T stage (T3–T4 vs. lower), and extranodal extension. Lower δ^13^C values in surgical margins were independently associated with an increased risk of disease recurrence (shorter DFS), after adjustment for established clinicopathological factors.

**Figure 5:**
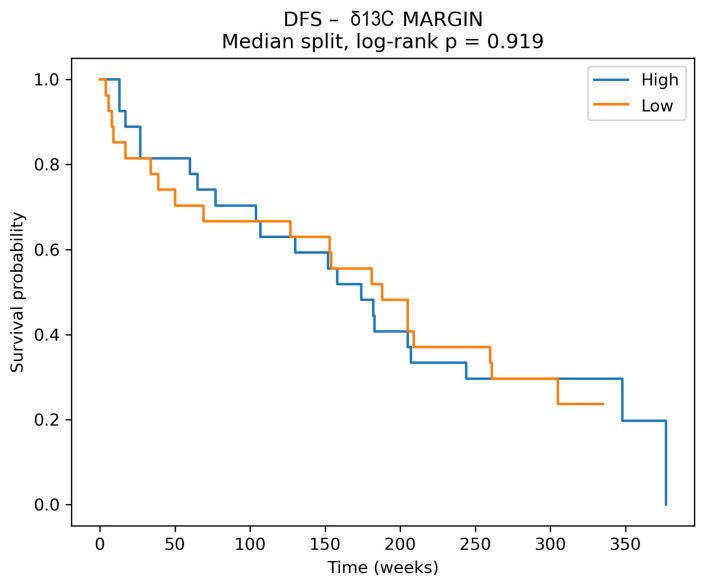
Disease-free survival (DFS) stratified by δ^13^C values in surgical margins. Kaplan–Meier curves comparing patients with high versus low δ^13^C levels in margin tissue, dichotomized at the median. No significant difference in DFS was observed between groups (log-rank *p* = 0.919), indicating a lack of prognostic discrimination for margin δ^13^C when analyzed as a dichotomous variable.

Among the evaluated biomarkers, δ^13^C measured in surgical margins emerged as a strong predictor of disease-free survival. Lower δ^13^C values in samples derived from surgical margin were associated with a significantly increased risk of disease recurrence or death (HR 5.66, 95% CI 2.05–15.63, *p* = 0.0008) and this association remained statistically significant after correction for multiple testing (false discovery rate-adjusted *p* = 0.0098) (Figure 4). In contrast, Kaplan–Meier analysis using a median-based cut-off did not demonstrate a statistically significant separation of survival curves (Figure 5, log-rank *p* = 0.919). This indicates that the prognostic signal of margin δ^13^C becomes apparent primarily when modeled as a continuous variable and after adjustment for clinicopathological factors rather than after dichotonization.

The apparent discrepancy between the non-significant Kaplan–Meier analysis and the significant multivariable Cox model most likely reflects information loss after median dichotomization and the fact that the adjusted continuous model better captures the prognostic gradient of margin δ^13^C.

**Figure 6:**
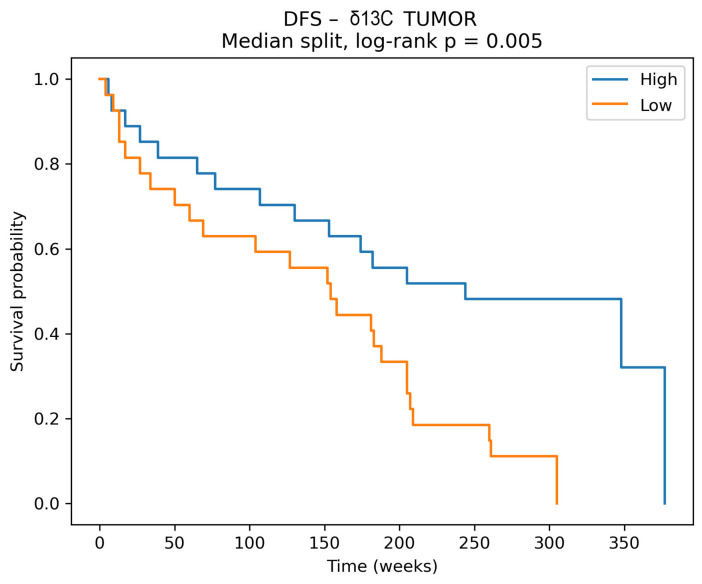
Disease-free survival (DFS) stratified by tumor δ^13^C levels. Kaplan–Meier curves comparing patients with high versus low δ^13^C values in tumor tissue, dichotomized at the median. DFS was defined as the time from primary surgery to recurrence (local, regional, or distant) or death. Patients without events were censored at the last follow-up. A significant difference in DFS between groups was observed (log-rank *p* = 0.005), with higher tumor δ^13^C associated with improved disease-free survival.

Interestingly, δ^13^C measured in tumor tissue showed only a nonsignificant trend toward shorter DFS (HR 1.44, 95% CI 0.97–2.14, *p* = 0.07), while δ^13^C tumor–margin differences were not associated with DFS. No robust associations were observed between δ^13^C and overall survival (OS). In contrast, Kaplan–Meier analysis for δ^13^C measured in tumor tissue demonstrated a statistically significant difference in disease-free survival between groups stratified at the median, with higher δ^13^C values associated with improved outcomes (Figure 6, log-rank *p* = 0.005). The significant Kaplan–Meier separation observed for tumor δ^13^C should be interpreted cautiously, as it was based on median dichotomization and unadjusted group comparison. In contrast, the multivariable Cox model assessed δ^13^C as a continuous predictor while accounting for major clinicopathological covariates. The lack of independent significance in the adjusted model suggests that the apparent Kaplan–Meier effect may reflect information loss due to dichotomization, residual confounding, and limited statistical power rather than a robust independent prognostic association.

Other spectrometric parameters, including percentage mass content of carbon and nitrogen and the [N]/[C] ratio, did not demonstrate consistent or statistically significant associations with DFS or OS across any tissue compartment.

These findings identify margin δ^13^C as a candidate biomarker for recurrence risk, distinct from tumor-based biomarkers associated with nodal metastasis.

**Figure A1:**
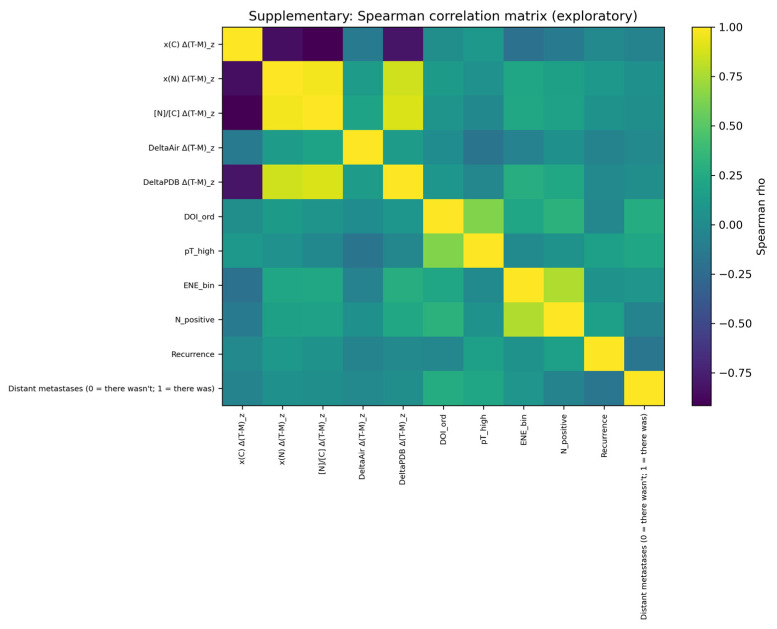
Spearman correlation matrix of spectrometric and clinicopathological variables (exploratory analysis). Heatmap illustrating pairwise Spearman rank correlation coefficients (ρ) between tumor–margin differences (Δ[T–M]) in spectrometric biomarkers and selected clinicopathological variables, including depth of invasion, pathological T stage, extranodal extension, lymph node status, recurrence, and distant metastases.

**Figure A2:**
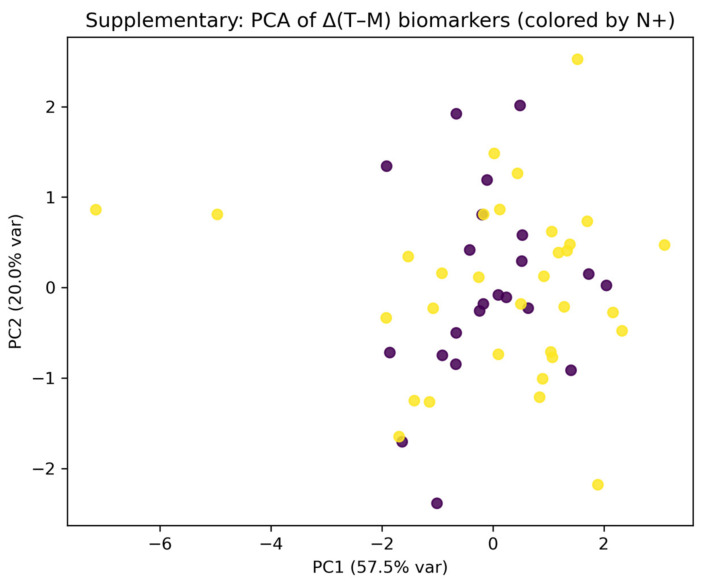
Principal component analysis (PCA) of tumor–margin spectrometric differences. Scatter plot of the first two principal components (PC1 and PC2), explaining 57.5% and 20.0% of total variance, respectively. Each point represents an individual patient, colored according to lymph node metastasis status (N0 vs. N+). PCA was performed on standardized Δ(T–M) spectrometric biomarkers. No clear clustering or separation between patients with and without lymph node metastases was observed, suggesting limited discriminative structure in the multivariate spectrometric profile at the exploratory level.

To contextualize these findings, additional exploratory analyses were conducted to examine the internal structure of tumor–margin spectrometric differences. Spearman correlation analysis revealed non-random correlations among Δ-biomarkers, indicating shared variance and structured relationships (Figure A1). Strong correlations were observed among spectrometric parameters, particularly between nitrogen-related variables and δ-values, whereas correlations between spectrometric biomarkers and clinical outcomes were generally weak. This analysis is exploratory and hypothesis-generating. Principal component analysis demonstrated that the first two components accounted for approximately 78% of the total variance, without clear separation according to lymph node status. Unsupervised clustering based on Δ-biomarkers identified two clusters with largely overlapping clinicopathological characteristics and outcome rates (Figure A2).

Together, these exploratory analyses suggest that tumor–margin spectrometric differences reflect a structured metabolic dimension within the dataset, while only selected biomarkers—most notably δ^13^C measured in surgical margins—show potential associations with clinical outcomes. Additional exploratory analyses are presented in the Appendix A.

## 4. Discussion

In this prospective study, we identified a reproducible and directionally consistent alteration of spectrometric signatures between tumor tissue and corresponding surgical margins. The percentage mass content of carbon was significantly lower in samples derived from oral carcinoma. The opposite pattern was observed for nitrogen content. Tumors were also characterized by lower δ^13^C and δ^15^N values, and increased nitrogen-to-carbon ratios relative to adjacent margins. These differences persisted after false discovery rate correction, indicating a robust metabolic shift. When focusing on a prognostic perspective, two findings deserve particular emphasis. First, tumor δ^15^N independently improved prediction of lymph node metastasis beyond established clinicopathological variables. The lack of a statistically significant univariate association for δ^15^N TUMOR should not be interpreted as contradicting its contribution in the multivariable model. Univariate analyses assess marginal differences between N0 and N+ groups and do not account for the clinical structure of the cohort. In contrast, the penalized multivariable model evaluates whether δ^15^N provides additional information beyond DOI and pT. The observed increase in AUC suggests that tumor δ^15^N may reflect a metabolic dimension of nodal dissemination not fully captured by anatomical tumor extent. Although tumor δ^15^N improved model discrimination, the magnitude of AUC change was modest. This does not by itself establish clinical usefulness, as discrimination alone is insufficient to determine whether a biomarker meaningfully improves decision-making in practice. Accordingly, the present findings should be viewed as hypothesis-generating and indicative of potential complementary prognostic value, requiring validation in larger cohorts with formal assessment of clinical utility. Second, margin δ^13^C emerged as a strong and independent predictor of disease-free survival (DFS), even after multiple testing correction. These findings suggest that isotopic composition reflects distinct biological dimensions of tumor aggressiveness. It can be assumed that, nitrogen-related tumor metabolism may be associated with regional metastatic potential, whereas carbon isotopic composition in surgical margins may reflect a field effect relevant to recurrence risk.

Our spectrometric data complement earlier findings on cancer-associated metabolic reprogramming, which has been extensively documented since Warburg’s original observations. Metabolomic studies demonstrate enhanced glutamine metabolism, nitrogen redistribution, and anabolic reprogramming in head and neck cancers [21,22,23,24,25,26]. Glutamine-derived nitrogen is essential to nucleotide synthesis, amino acid production, and redox balance in proliferating tumor cells. Although tumor δ^15^N improved discrimination of nodal metastasis beyond clinicopathological variables, its lack of independent association with DFS and OS suggests that nitrogen isotopic alterations may reflect early metastatic propensity rather than determinants of long-term survival. Given the modest sample size and limited number of events, findings should be interpreted as hypothesis-generating and require external validation. Alterations in δ^15^N values have been reported in various malignances and are thought to reflect changes in nitrogen associated with rapid cellular proliferation. Our observation of elevated mean percentage tumor mass content of N and association of δ^15^N values with lymph node metastasis is biologically plausible. Altered nitrogen fractionation may reflect intensified anabolic processes and metabolic plasticity required for metastatic potential [27,28].

A mechanistic explanation for the observed δ^15^N depletion in tumor tissue can be derived from recent advances in isotope biochemistry of cancer metabolism. Nitrogen isotope composition reflects the balance between nitrogen influx, primarily via glutaminolysis, and nitrogen efflux through urea, ammonium, and amino acid release. Enzymatic reactions involved in these pathways exhibit kinetic isotope effects that preferentially discriminate against ^15^N, leading to the accumulation of ^15^N-depleted nitrogen pools within tumor cells.

In particular, urea cycle activity contributes to isotopic fractionation, as key enzymatic steps—including glutaminase and carbamoyl phosphate synthetase—generate ^15^N-depleted intermediates, while excretion of urea further reinforces this effect. Consequently, δ^15^N values may represent an integrated readout of tumor nitrogen fluxes and metabolic reprogramming rather than a single pathway-specific alteration [29,30].

Although the primary prognostic associations in the present study were driven by nitrogen isotope parameters, carbon isotope composition remains an important component of the metabolic profiling. Tumor tissue exhibited lower δ^13^C values compared with margins. Malignant tissues frequently demonstrate relative 13C depletion, potentially due to preferential substrate utilization and altered lipid biosynthesis. More importantly, margin δ^13^C strongly predicted DFS. This finding may be interpreted in the context of field cancerization in head and neck cancer, as refined by contemporary molecular and genomic studies demonstrating genetically and metabolically altered mucosal fields surrounding primary OSCC lesions [31,32,33]. Stable isotope shifts in histologically negative margins may therefore reflect subclinical metabolic reprogramming within the tumor microenvironment [34].

Another important aspect of our findings concerns survival aspect and potential spectrometric biomarkers. Despite the association between tumor δ^15^N and nodal metastasis, δ^15^N did not independently predict DFS or OS after adjustment. Instead, depth of invasion and pathological T category remained dominant determinants of survival, consistent with the established literature [13,35]. These results suggest that isotopic biomarkers may primarily reflect early metastatic potential rather than long-term survival, which is influenced by multiple clinical and treatment-related factors.

An additional aspect that requires consideration when interpreting isotopic biomarkers is the potential influence of individual variability and environmental factors, particularly diet, on tissue isotope composition. Stable isotope ratios in human tissues partly reflect long-term dietary intake. Consequently, inter-individual differences in δ^13^C or δ^15^N values in analyzed tissue samples may also theoretically arise from dietary patterns rather than tumor biology alone. On the other hand, similar ^15^N depletion patterns have been observed in both tumor tissues and several cultured cancer cell lines (breast, colorectal and prostate cancer), despite differences in nutrient availability and systemic influences [36,37]. This supports the interpretation that isotopic signatures primarily reflect intrinsic tumor metabolism rather than dietary variability [38].

Several features of the present study design reduce the likelihood that the observed findings are driven primarily by dietary variability. First of all, the paired sampling strategy allowed each patient to serve as their own internal control. Tumor tissue was directly compared with the corresponding surgical margin obtained during the same procedure, thereby minimizing the impact of systemic or lifestyle-related isotopic differences between individuals. Secondly, the tumor–margin comparisons demonstrated a consistent and directionally uniform pattern across the cohort, with tumor tissues showing lower δ^13^C and δ^15^N values and higher nitrogen-to-carbon ratios compared with margins. The statistical significance of these differences remained robust after correction for multiple testing, indicating that the observed isotopic shifts represent a reproducible biological signal rather than isolated observations.

Importantly, the prognostic association identified in this study was not driven by extreme individual measurements. Sensitivity analyses confirmed that the observed relationships were stable and not dependent on single outlying observations within the dataset. Instead, the association between isotopic composition and clinical outcomes emerged from the overall distribution of measurements across patients.

Taken together, these considerations suggest that the isotopic alterations observed in OSCC tissue most likely reflect metabolic reprogramming associated with tumor biology rather than purely dietary effects. Nevertheless, stable isotope signatures should be interpreted as integrated biological phenotypes that may capture both systemic environmental exposures and tumor-specific metabolic processes.

From clinical perspective, the identification of metabolic biomarkers associated with survival and recurrence is of particular importance in OSCC, where surgical decisions and adjuvant therapy selection significantly impact patient quality of life.

Tumor δ^15^N may serve as an adjunct metabolic biomarker for nodal risk stratification, while margin δ^13^C may help identify patients at increased risk of recurrence despite histologically clear margins. Isotope ratio mass spectrometry offers reproducibility and relatively low analytical complexity compared with high-throughput metabolomics platforms. Moreover, IRMS-based isotopic analysis requires only small amounts of tissue and does not interfere with routine histopathological assessment. Therefore, it could potentially be integrated into existing diagnostic workflows as an adjunctive tool for risk stratification.

Although comprehensive metabolomic and genomic profiling provide broader molecular information, their routine clinical implementation remains limited by analytical complexity, data integration requirements, cost, and incomplete standardization across platforms. In contrast, IRMS-based isotopic profiling does not aim to replace multi-omic approaches but to provide a simpler tissue-level readout of integrated metabolic reprogramming. Its potential incremental value lies in capturing a cumulative biochemical phenotype from small surgical specimens, using analytically robust measurements that may be easier to standardize and incorporate into routine pathology workflows. Therefore, isotopic profiling should be interpreted as a complementary biomarker strategy, rather than a competing alternative to genomic or metabolomic profiling.

In the future, isotopic profiling may help identify patients at higher risk of poor outcomes who could benefit from intensified surveillance or adjuvant treatment, as well as patients with favorable metabolic profiles who might avoid overtreatment. Isotope analysis could complement existing prognostic tools in oral cancer research.

Despite its strengths which include the prospective design, paired sampling strategy, strict endpoint definitions, and penalized multivariable modeling, the present study has several limitations. Limitations include small sample size, cohort skewed toward advanced-stage disease, single-center setting, and lack of integrated molecular analyses. While IRMS captures the net isotopic outcome of metabolic flux, concurrent expression analysis of key enzymes (e.g., glutaminase 1) was not performed and remains a subject for future investigation.

Due to the relatively small sample size and limited number of events, all multivariable analyses were deliberately restricted in complexity in accordance with the pre-specified statistical analysis plan. Penalized regression techniques and bootstrap internal validation were applied to mitigate overfitting; however, the findings should be interpreted as hypothesis-generating and require validation in larger, independent cohorts.

The study cohort was predominantly composed of patients with advanced-stage disease, reflecting a tertiary referral surgical population. Therefore, the generalizability of the findings to early-stage OSCC remains limited and should be interpreted with caution.

Future studies integrating isotopic analysis with molecular and metabolic assays, including enzyme expression and metabolic flux measurements, are warranted.

To sum up, this manuscript suggests plausible but still preliminary clinical applications of IRMS parameters. Although the findings do not yet support using isotopic profiling as a stand-alone decision-making tool in routine care, they do indicate a potential role as an adjunct biomarker strategy for postoperative risk stratification.

Tumor δ^15^N could be used as an additional biological signal of metastatic propensity, complementing conventional clinicopathological factors such as DOI and pT category, provided that the association is confirmed in larger external cohorts.

A second, and perhaps more clinically intriguing, application is identification of patients at increased risk of recurrence despite histologically negative margins. In practical terms, such patients might in the future be candidates for closer surveillance, more cautious postoperative risk assessment, or inclusion in intensified monitoring strategies. However, this remains an exploratory signal rather than an established clinical test.

A third realistic application is in the development of multivariable prognostic models that integrate metabolic information with standard pathology. IRMS-based isotopic profiling is not intended to replace metabolomics or genomic profiling, but rather to provide a simpler and potentially more standardized readout of integrated metabolic reprogramming from small tissue samples. In that sense, the main clinical value of the present findings may lie in contributing an additional layer of biologically meaningful information to combined prognostic models, rather than functioning as an isolated biomarker.

Overall, the results of this study support a future clinical role for isotopic profiling as an adjunctive postoperative risk stratification tool, particularly for identifying biologically aggressive disease not fully captured by conventional histopathological variables. However, at the present stage, these applications remain translational and investigational rather than practice-changing.

## 5. Conclusions

Stable isotope spectrometric profiling captures biologically meaningful metabolic alterations in OSCC. Tumor δ^15^N is associated with lymph node metastasis, whereas margin δ^13^C independently predicts disease-free survival. These findings support further translational validation of isotopic biomarkers in oral oncology.

## Figures and Tables

**Table 1:** Baseline clinicopathological characteristics of the study group.

	Overall Cohort (*N* = 54)
**Patient Characteristics**	
Age, years, median (IQR)	68.0 (60.2–71.0)
Gender, *n* (%)	
Male	33 (61.1%)
Female	21 (38.9%)
Ever smoker, *n* (%)	33 (61.1%)
Smoking intensity, pack-years, median (IQR)	20.0 (0.0–30.0)
Alcohol consumption, *n* (%)	16 (29.6%)
**Tumor Characteristics**	
Tumor site, *n* (%)	
Lower gingiva	21 (38.9%)
Floor of the mouth	18 (33.3%)
Tongue	12 (22.2%)
Buccal mucosa	3 (5.6%)
pT category, *n* (%)	
T4	26 (48.1%)
T3	15 (27.8%)
T2	11 (20.4%)
T1	2 (3.7%)
pN category, *n* (%)	
N3b	26 (48.1%)
N0	22 (40.7%)
N2b	3 (5.6%)
N1	2 (3.7%)
N2c	1 (1.9%)
Clinical Stage, *n* (%)	
IV	43 (79.6%)
III	5 (9.3%)
II	4 (7.4%)
I	2 (3.7%)
**Pathology**, *n* (%)	
Grade	
2	35 (64.8%)
3	10 (18.5%)
1	9 (16.7%)
DOI (depth of infiltration)	
DOI > 10 mm	29 (53.7%)
DOI 5.1–10 mm	23 (42.6%)
DOI 1–5 mm	2 (3.7%)
Extranodal extension (ENE)	25 (46.3%)
Angioinvasion and/or neuroinvasion	25 (46.3%)
Angioinvasion	20 (37.0%)
Neuroinvasion	17 (31.5%)
**Lymph Node Metrics**	
Resected lymph nodes, median (IQR)	34 (26–42)
Resected metastatic lymph nodes, median (IQR)	1 (0–4)
Lymph node ratio, median (IQR)	0.09 (0.05–0.15)
**Oncologic Outcomes,** *n* (%)	
Locoregional recurrence	20 (37.0%)
Distant metastases	13 (24.1%)
Deaths	34 (63.0%)

**Table 2:** Paired comparison of spectrometric biomarkers between tumor tissue and surgical margins.

Biomarker	Median TUMOR	Median MARGIN	Median Δ(T–M)	Δ 95% CI Lower	Δ 95% CI Upper	Wilcoxon *p*	*p*_FDR
Carbon (%)	46.21	51.11	−4.24	−5.42	−2.24	0.000	0.000
Nitrogen (%)	12.65	10.47	1.59	0.95	2.43	0.000	0.000
[N]/[C]	0.27	0.21	0.05	0.03	0.07	0.000	0.000
δ^15^N (‰)	8.75	9.64	−0.77	−0.96	−0.52	0.000	0.000
δ^13^C (‰)	−22.40	−23.34	0.67	0.34	1.13	0.000	0.000

Values are presented as medians. Differences between paired tumor and margin samples were assessed using the Wilcoxon signed-rank test. Effect sizes are expressed as median paired differences (Δ[TUMOR–MARGIN]) with corresponding 95% confidence intervals. *p* values were adjusted for multiple comparisons using the Benjamini–Hochberg false discovery rate (FDR) method.

**Table 3:** Univariate analysis of spectrometric parameters and lymph node metastasis.

Biomarker	Sample	N0 Median (IQR) *n* = 22	N+ Median (IQR) *n* = 32	MWU *p*	OR Per 1 SD	OR 1SD 95% CI Lower	OR 1SD 95% CI Upper	Logit *p* (OR)	MWU *p*_FDR
Nitrogen (%)	TUMOR	0.31 (−0.01–0.41)	0.41 (0.22–0.52)	0.09	1.27	0.74	2.19	0.39	0.45
Nitrogen (%)	Δ(T–M)	−0.08 (−0.53–0.36)	0.37 (−0.50–0.75)	0.21	1.13	0.66	1.93	0.67	0.45
[N]/[C]	TUMOR	0.35 (0.10–0.41)	0.40 (0.26–0.51)	0.19	1.14	0.67	1.95	0.63	0.45
[N]/[C]	Δ(T–M)	−0.18 (−0.41–0.36)	0.40 (−0.58–0.73)	0.18	1.09	0.64	1.87	0.75	0.45
δ^15^N (‰)	TUMOR	−0.31 (−0.90–0.32)	0.19 (−0.70–0.86)	0.17	1.35	0.77	2.37	0.30	0.45
δ^15^N (‰)	MARGIN	−0.31 (−1.06–0.33)	0.09 (−0.24–0.56)	0.13	1.29	0.73	2.28	0.38	0.45
δ^13^C (‰)	Δ(T–M)	0.02 (−0.05–0.13)	0.25 (−0.10–0.41)	0.11	1.70	0.59	4.89	0.32	0.45
δ^13^C (‰)	TUMOR	0.19 (−0.23–0.34)	0.32 (−0.13–0.73)	0.28	1.13	0.66	1.93	0.67	0.52
Carbon (%)	Δ(T–M)	−0.07 (−0.33–0.31)	−0.30 (−0.63–0.35)	0.39	1.00	0.58	1.72	0.99	0.66
Carbon (%)	TUMOR	−0.34 (−0.52–−0.28)	−0.35 (−0.47–−0.23)	0.58	0.98	0.57	1.68	0.94	0.87
Carbon (%)	MARGIN	−0.11 (−0.73–0.31)	0.01 (−0.65–0.43)	0.79	0.98	0.57	1.68	0.94	0.88
[N]/[C]	MARGIN	0.03 (−0.73–0.72)	−0.29 (−0.45–0.65)	0.83	1.04	0.61	1.79	0.88	0.88
δ^15^N (‰)	Δ(T–M)	−0.07 (−0.72–0.40)	0.13 (−0.67–0.61)	0.76	1.09	0.63	1.87	0.76	0.88
δ^13^C (‰)	MARGIN	−0.02 (−0.39–0.19)	−0.12 (−0.36–0.07)	0.77	0.66	0.28	1.58	0.35	0.88
Nitrogen (%)	MARGIN	0.18 (−0.74–0.68)	−0.07 (−0.44–0.75)	0.95	1.14	0.66	1.97	0.63	0.95

*n*—number of patients. Median and IQR values of IRMS biomarkers were standardized by subtracting the cohort mean and dividing by standard deviation. Positive and negative values indicate measurements above and below the mean.

**Table 4:** Multivariable prediction of lymph node metastasis (N+) using clinical and spectrometric variables.

Biomarker	OR Per 1 SD (Penalized) (95% CI)	AUC	ΔAUC vs. Base
Clinical base model (DOI + pT)	—	0.690	0.000
δ^15^N TUMOR_z	1.179 (0.806–2.265)	0.717	0.027
δ^15^N Δ(T-M)_z	0.940 (0.541–1.640)	0.709	0.018
[N]/[C] Δ(T-M)_z	1.088 (0.655–1.973)	0.696	0.006

## Data Availability

The data on which this study is based will be made available upon request at https://www.researchgate.net/profile/Katarzyna-Bogusiak (accessed on 28 April 2026).

## Abbreviations

The following abbreviations are used in this manuscript:

OSCC	Oral Squamous Cell Carcinoma
DSS	Disease-Specific Survival
ENE	Extranodal extension
DOI	Depth of invasion
LVI	Lymphovascular invasion
PNI	Perineural invasion
EGFR	Epidermal Growth Factor Receptor
VEGF	Vascular Endothelial Growth Factor
IRMS	Isotope Ratio Mass Spectrometry
PDB	Pee Dee Belemnite
OS	Overall survival
AUC	Area under the curve
AJCC	American Joint Committee on Cancer
FDR	False discovery rate
ROC	Receiver Operating Characteristic
